# More resistant tendons obtained from the association of *Heteropterys aphrodisiaca *and endurance training

**DOI:** 10.1186/1472-6882-11-51

**Published:** 2011-06-28

**Authors:** Juliana C Monteiro, Marcos LM Gomes, Tatiana C Tomiosso, Wilson R Nakagaki, Mariana M Sbervelheri, Danilo L Ferrucci, Edson R Pimentel, Heidi Dolder

**Affiliations:** 1 Departamento de Ciências Agrárias e Biológicas, Centro Universitário Norte do Espírito Santo, Universidade Federal do Espírito Santo, BR 101 Norte - Km 60, CEP 29932-540, São Mateus, ES, Brasil; 2 Departamento de Biologia Estrutural e Funcional, Instituto de Biologia, Universidade Estadual de Campinas, CEP 13083-863, CP 6109, Campinas, SP, Brasil; 3 Departamento de Histologia, Instituto de Ciências Biomédicas, Universidade Federal de Uberlândia, CEP 38400-902, CP 592, Uberlândia, MG, Brasil; 4 Departamento de Bioquímica, Instituto de Biologia, Universidade Estadual de Campinas, CEP 13083-863, CP 6109, Campinas, SP, Brasil

## Abstract

**Background:**

Popular Brazilian medicine uses *Heteropterys aphrodisiaca *infusion as a tonic or stimulant, for the treatment of nervous debility and breakdown and for muscle and bone weakness. This study investigated the effects of *Heteropterys aphrodisiaca *infusion on the tendon properties and extracellular matrix of rats under endurance training.

**Methods:**

Wistar rats were grouped as follows: CS- control sedentary, HS- *H. aphrodisiaca *sedentary, CT-control trained, HT- *H. aphrodisiaca *trained. The training protocol consisted in running on a motorized treadmill, five times a week, with weekly increase in treadmill speed and duration. Control groups received water while the HS and HT groups received *H. aphrodisiaca *infusion, daily, by gavage for the 8 weeks of training. Achilles tendons were frozen for biochemical and biomechanical analysis or preserved in Karnovsky's fixative, then processed for histomorphological analysis with light microscopy.

**Results:**

Biomechanical analysis showed significant increase in maximum load, maximum stress, modulus of elasticity and stiffness of the HT animals' tendons. The metalloproteinase-2 activity was reduced in the HT group. The compression region of HT animals' tendons had a stronger and more intense metachromasy, which suggests an increase in glycosaminoglycan concentration in this region of the tendon. The most intense birefringence was observed in both compression and tension regions of HT animals' tendons, which may indicate a higher organizational level of collagen bundles. The hydroxyproline content increased in the HT group.

**Conclusions:**

The association of endurance training with *H. aphrodisiaca *resulted in more organized collagen bundles and more resistant tendons to support higher loads from intense muscle contraction. Despite the clear anabolic effects of *Heteropterys aphrodisiaca *and the endurance exercise association, no side effects were observed, such as those found for synthetic anabolic androgenic steroids.

## Background

*Heteropterys aphrodisiaca *O. Mach. (Malpighiaceae), also known as "nó-de-cachorro", "nó-de-porco" and "cordão-de-São-Francisco", was described by Hoehne in 1920 as a plant with stimulative and aphrodisiac properties. Also, Brazilian traditional medicine uses *H. aphrodisiaca *root infusion as a tonic or stimulating treatment and for nervous debility, nervous breakdown and for muscle and bone weakness. This plant is found mainly in the "Cerrado" regions (a savanna-like biome) of Mato Grosso and Goiás States (Brazil) [1,2].

Previous studies with *H. aphrodisiaca *suggested that the root extract could increase corporal and testicular weight, as well as Leydig cell volume within the rat testis [3,4]. Leydig cells produce testosterone, thus regulating muscle protein metabolism, erythropoiesis, plasma lipids, bone metabolism and cognitive functions [5]. Since testosterone regulates growth, structure and functions of accessory sex organs [6] and the treatment with *H. aphrodisiaca *infusion showed no alterations of these organs' weights, Chieregato [3] suggested this hormone resulted in weight gain since it acts on the skeletal muscle mass.

Higher doses of anabolic androgenic steroids (AAS), when combined with a training program, have been claimed to increase muscle strength and mass [7]. However, some reports have mentioned that tendons do not follow the same rate of protein gain as achieved by the muscle bulk, and therefore high intensity and frequency of training might lead to tendon ruptures [8,9]. Some studies using animals suggested that steroids modify the collagen crimp pattern and tendon biomechanical properties [10]. Steroid use may cause collagen dysplasia in the flexor digitorum tendon of mice [11], increasing tendon stiffness and diminishing both elongation and energy absorption [12,13]. Thus, such tendons are more likely to fail during strenuous activities. In addition, AAS treatment can impair tendon tissue remodeling by down regulating matrix metalloproteinase (MMP) activity in animals undergoing physical exercise, hence increasing the potential for tendon injury [14]. In human beings, morphological changes at the muscle-tendon junction, as well as tendon ruptures were found when anabolic steroids were associated with exercise [15]. High intensity loads during exercise seem to play a role in the process that allows the deleterious effects of steroids to be manifested. In the above studies, the groups that had been treated with both steroids and exercise suffered the worst injuries.

The response of tendons to exercise and some treatments may be analyzed regarding structural, chemical and mechanical aspects. However, most studies have been limited to analysis of only one or two of these aspects [16]. The present study aggregates biochemical, structural and biomechanical data in order to precisely investigate the effects of *H. aphrodisiaca *infusion on the tendon properties under long term endurance training. This study is part of a comprehensive study dealing with the effects of *H. aphrodisiaca *administration and intensive endurance exercise on some biological systems.

## Methods

### Animals

Adult Wistar rats, 90 days old, were obtained from the Center for Biological Investigation - CEMIB (State University of Campinas, Campinas, SP, Brazil). The rats were housed, three per cage, under standard conditions with 12 h L:12 h D cycle. Animals were provided with commercial rat feed and water *ad libitum*. The Institutional Committee for Ethics in Animal Care and Use of this University approved the experimental protocol (process n° 1233-1).

### Medicinal Plant

*H. aphrodisiaca *roots were collected in February 2007, in Mato Grosso State, Brazil. The species was identified by comparison with the voucher herbarium specimen of the plant at the Herbarium of the Mato Grosso Federal University, Brazil (number 23928). The roots were dried at room temperature, crushed and powdered using a grinding mill. The infusion was routinely prepared by pouring 100 mL of boiling water over 25 g of powdered roots, which was allowed to steep for 4 hours, then filtered by using filter paper. The yield was an infusion of 68.66 mg of dry extract (6.866% w/v) and a yield of 6.832% (w/w) in terms of initial crude dry weight of plant material. The infusion was prepared every four days and stored in the refrigerator. The doses of *H. aphrodisiaca *were selected according to previous studies [4].

### Study groups and experimental protocol

Forty-eight male rats were divided into four groups (n = 12/group): two sedentary (CS and HS) and two submitted to involuntary running on a motorized treadmill (CT and HT). All groups received either 0.5 mL of distilled water (CS and CT, control groups) or *H. aphrodisiaca *infusion (HS and HT). Either water or infusion (104 mg/day) was administered by gavage during the 8 weeks of both training and sedentary periods. Trained rats (group CT and HT) were allowed to adapt to treadmill running for a 3 week period, prior to the beginning of the experimental protocol, which consisted of low to moderate level exercise carried out daily for 5 days a week (Table 1). After adaptation, trained rats were subjected to 8 weeks of intensive aerobic exercise training (treadmill running), also on a weekly cycle of 5 consecutive exercising days followed by a two day rest, as shown in Table 1, adapted from [17-20]. This program is a form of endurance training and does not compare with power training [18]. Forty-eight hours after the last training, the rats were anesthetized with xylazine chloride (Anasedan, Vetbrands, São Paulo, Brazil) and ketamine chloride (Cetamin, Syntec, Cotia, Brazil) (5 and 80 mg/Kg body weight, respectively). The right and left tendons were excised and frozen for biochemical, biomechanical and zymographical analysis or preserved in Karnovsky's fixative for morphological analysis.

**Table 1:** Exercise protocol in treadmill running

Event	Week	Velocity (m/min)	Duration (min)
Treadmill	1	10.68	5
adaptation	2	12.42	7,5
	3	14.16	10

	1	14.16	20
Training	2	19.62	30
	3	19.62	40
	4-8	22.92	45

### Light Microscopy Analysis

Tendon samples (n = 4/group) were immersed in Karnovsky's fixative for 24 h and then processed for paraffin embedding (Histosec, Merck). Longitudinal serial sections, 7 μm thick, were stained with toluidine blue (0.025%) in McIlvaine buffer (0.03 M citric acid, 0.04 M sodium phosphate, dibasic - pH 4.0) and observed by polarized and conventional light microscopy. The organization of collagen bundles was examined with a Nikon E800 microscope (polarized light microscopy), connected with Cool Snap Pro-Color camera (Media Cibernetica). Polarized light microscopy of a highly ordered fiber, such as collagen, shines brightly. To assess birefringence, the analyzer and the polarizer were crossed and the material was positioned at an angle of 45° relative to the polarizer. Proteoglycans were detected in extracellular matrix tissue sections stained with Toluidine Blue (pH 4.0) and observed under an Olympus BX 41 light microscope (conventional light microscopy). Since proteoglycans have many closely placed negative radicals, sections with high concentrations of proteoglycans, stained with toluidine blue, determine closely arranged and interacting molecules of this stain, replacing the blue with a violet coloring.

### Extraction procedures

The tendons (n = 4/group) were treated with 25 volumes of 4 M guanidine chloride (GuHCl), containing 1 mM phenylmethylsulfonyl fluoride (PMSF) and 20 mM ethylenediamine tetraacetic acid (EDTA) in 50 mM sodium acetate buffer, pH 5.8 (Heinegård and Sommarin, 1987) at 4°C for 24 h. The mixture was then centrifuged (20,000 × g, 4°C, 30 min), the supernatant precipitated in acetate-ethanol and used in the biochemical analyses.

### Electrophoresis (SDS-PAGE)

SDS-PAGE was performed according to Zingales [21], using gradient gels (4-16%). The tendon extracts (50 μL) from all experimental groups were precipitated with 100 μL of 50 mM acetate buffer, pH 7.4, and 9 volumes of absolute ethanol, for 24 hr at 4°C. These samples were analyzed by SDS-PAGE (50 μL). Gels were stained with Coomassie Brilliant Blue R-250. The relative molecular masses were estimated by comparisons with protein standard molecular mass markers.

### Quantitative analysis

The amount of proteins in the extract of GuHCl was measured by the Bradford method [22], using bovine serum albumin (BSA) (1 mg/mL) as a standard. The readings were performed in a micro-plate reader at 595 nm. Sulfated glycosaminoglycans (GAG) of GuHCl extracts were quantified by the dimethylmethylene blue method (DMMB) [23] using chondroitin sulphate (1 mg/mL) as a standard, and readings were performed in a micro-plate reader at 526 nm. To quantify hydroxyproline, tendon fragments (n = 4) were dehydrated in acetone for 48 h and, subsequently, for another 24 hours in a mixture of chloroform and ethanol, at a ratio of 2:1. The tendon fragments were then hydrolyzed in 6 N HCl (10 mg of tissue/mL), for 18 h at 120°C, and the hydrolysate was neutralized with 6N NaOH. Samples were treated with chloramine T solution and perchloric acid/aldehyde, as described by Stegemann and Stalder [24]. After incubation for 15 min at 60°C, the material was cooled and absorbance was measured at 550nm in a spectrophotometer, Ultrospec 2100 (Pro Amersham Biosciences, England). The amount of hydroxyproline in the sample was calculated by comparison with a standard curve of hydroxyproline, and expressed as mg/g of wet tissue.

### Gelatin zymography

Tendon fragments (n = 4/group) were incubated in 0.3 mL of extraction buffer (10 mM cacodylic acid, pH 5.0, 0.15 M NaCl, 1 μM ZnCl_2_, 20 mM CaCl_2_, 1.5 mM NaN_3_, 0.01% Triton X-100 [v/v]), at 4°C for 24 hours. After this period, the solution was submitted to centrifugation for 10 minutes (20,000 × g at 4°C). Samples were dried and resuspended in the same extraction buffer (pH 5.0). Zymography assays were performed on 10% polyacrylamide electrophoresis gels containing 0.1% gelatin, using 8 μg and 50 μg of protein per sample to indentify MMP-2 and MMP-9, respectively. After electrophoresis, the gels were washed with 2.5% Triton X-100 at room temperature and incubated overnight in a solution of 50 mM Tris-HCl, pH 8.0, 5 mM CaCl_2 _and 0.02% NaN_3 _at 37°C for 20 hours. Finally, the gels were stained with Coomassie Brilliant Blue. The protein bands corresponding to gelatinolytic activity were observed after washing the gels with a solution containing 30% methanol and 10% acetic acid. The gel was evaluated by band densitometry using the Scion Image program. Each sample was analyzed individually and the experiments were repeated four times, with three gels used for MMP activity quantification while the fourth was incubated in EDTA (20 mM) to confirm the presence of MMPs. The total MMP-2 activity corresponds to the sum of each isoform's activity.

### Biomechanical test

After euthanasia, the right paw with the gastrocnemius and soleus muscles and Achilles tendon was excised. Five tendons of each group were used for the mechanical test. They were kept in physiological solution until tested, to prevent drying of the tissues. For the test, the specimens were clamped in a mechanical support with the myotendinous junction and the phalanges at opposite extremities, as employed by Nakagaki and colleagues [25]. The clamp-to-clamp distance was maintained at 7 mm for all tests. Each tendon was submitted to a pre-conditioning test, with 10 cycles of loading-unloading from 0 to 0.5 mm, at a speed of 20 mm/min [26] and then submitted to the uniaxial tensile test. During the test, the tendon was subjected to a gradual increase in load at a constant displacement rate of 20 mm/min using a load cell of 1 kN, until the tendon broke [26]. The data were digitized, displayed and stored in a computer. These data were used to calculate the structural properties (maximum load and displacement at maximum load) and the material properties (maximum stress, strain at maximum stress and modulus of elasticity or Young's modulus) of the tendons in each group [27]. The maximum stress was obtained from the relation between load (N) and cross-section area (CSA) (mm^2^) and expressed in MPa (megapascal). The CSA was determined according to Goodship and Birch [28]. Each tendon had its shape cast using an alginate dental impression paste (Avagel) manufactured by Dentsply. The CSA mould obtained was cut transversally and photographed. These images were then analyzed with the software Image Pro-Plus. The strain was calculated using the formula ε = ΔL/L_0_, where ΔL = L-L_0 _(L = final length and L_0 _= initial length). Strain was calculated based on clamp-to-clamp displacement. The elastic modulus of each group was measured in the interval corresponding to the most linear region of the stress-strain curve for each sample. The extrinsic stiffness was calculated as the slope of the linear region of the load-displacement curve (in N/mm). The experiments used a machine specifically designed for testing the mechanical properties of materials (MTS, model TETSTAR II) designed by the Laboratory of Mechanical Properties, School of Mechanical Engineering, UNICAMP.

### Statistical analysis

Statistica software (v 8.0) (Tulsa, OK, USA) was used for the statistical analysis. All data were presented as mean ± standard deviations (S.D.), and a value of p < 0.05 was considered significant. The statistical comparison among the control and treated groups was determined using one-way ANOVA followed by the post hoc test of Tukey. In addition, two-way ANOVA was used, when appropriate, to determine how *H. aphrodisiaca *treatment and/or exercise training affected the results, and whether there was interaction between these two conditions.

## Results

### Biochemical assay

**Figure 1:**
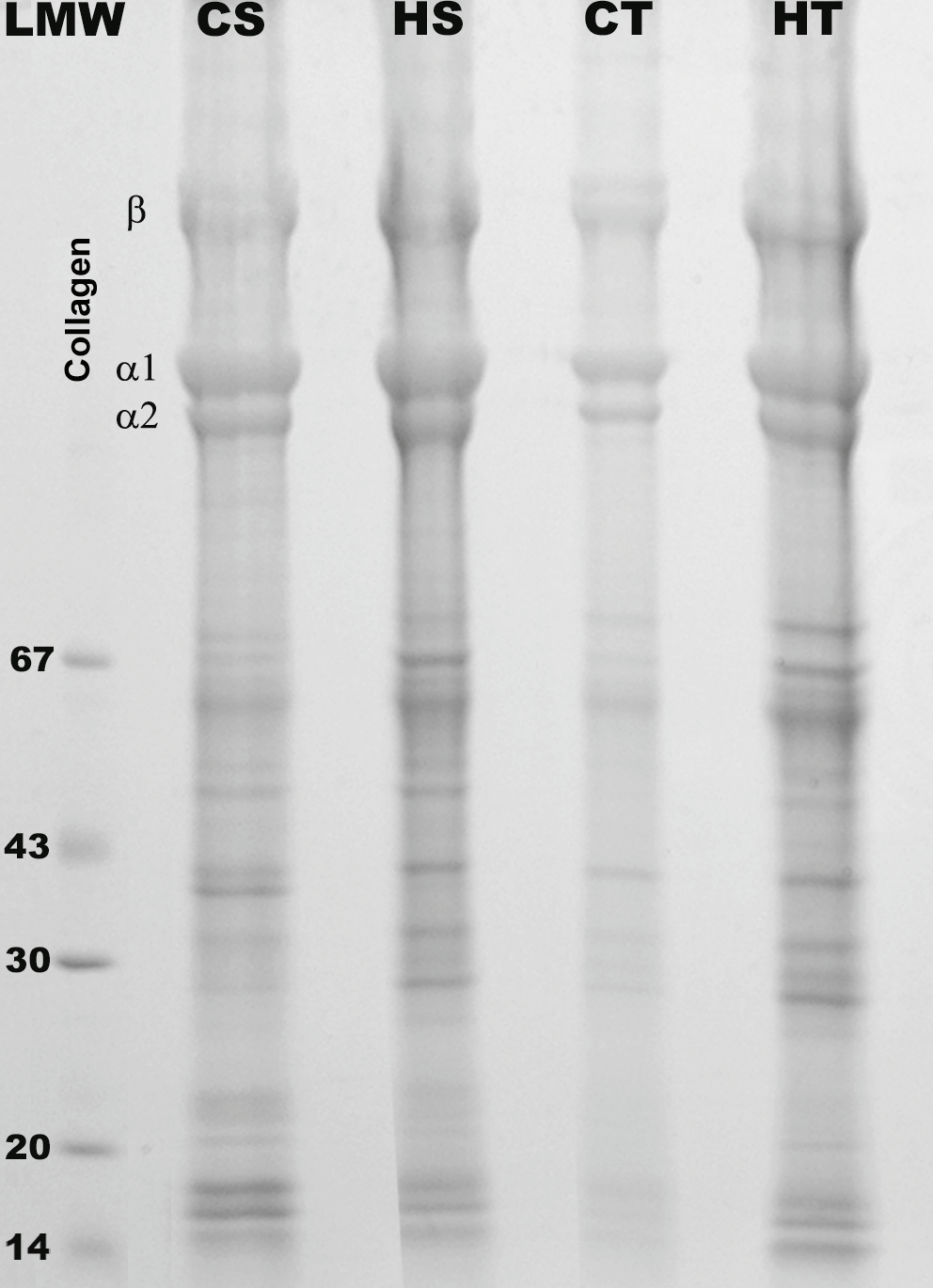
**SDS-PAGE of Guanidinium chloride extract of the Achilles tendon**. Analysis in SDS-PAGE showed faint bands of collagen and other proteins in control trained animals (CT). Note that *H. aphrodisiaca *treatment (HS) maintained the same bands displayed in control sedentary animals (CS) and also exhibited other NCP bands. In treated and exercised animals (HT) the appearance of NCP was accentuated. LMW- Low molecular weight standard.

Analysis in SDS-PAGE of the Guanidinium chloride extract showed the presence of faint bands of collagen and noncollagenous proteins (NCP) in extracts of animals of the CT group, compared with other groups. This result showed that exercise by itself markedly reduced the presence of collagen and NCP. However *H. aphrodisiaca *treatment in sedentary rats maintained the same bands as displayed in CS, and also a few additional ones. Furthermore, exercise associated with *H. aphrodisiaca *(HT group) accentuated the appearance of NCP (Figure 1).

**Figure 2:**
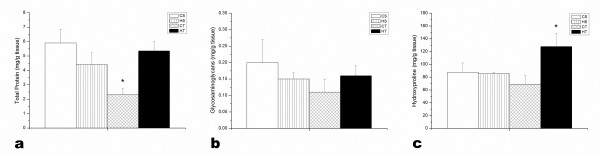
**Quantifications of noncollagenous protein (NCP), sulfated glycosaminoglycans and hydroxyproline in the Achilles tendon**. The NCP levels (a) were significantly decreased with exercise by itself, whereas *H. aphrodisiaca *treatment did not alter protein levels. The sulfated glycosaminoglycan levels (b) were statistically similar in the four groups. Observe the remarkable effect of training plus *H. aphrodisiaca *treatment on the hydroxyproline content (c). CS-control sedentary; HS- *H. aphrodisiaca *sedentary; CT- control trained; HT- *H. aphrodisiaca *trained. The columns are the mean ± SD. * Differences were significant for p < 0.05 (ANOVA) compared with other groups, by Tukey test.

The NCP levels, measured by protein analysis with the Bradford method, were significantly decreased (p < 0.05) in animals of the control trained group (CT) when compared to other groups (Figure 2a), confirming the SDS-PAGE analysis. Two-way ANOVA showed interaction between training and the treatment with plant infusion for tendon protein content (p = 0.0008). The sulfated glycosaminoglycan levels were statistically similar among all groups (Figure 2b).

Hydroxyproline is an indicator of collagen concentration in tissues. Its content was the highest in HT group (Figure 2c). Two-way ANOVA showed that there was interaction between endurance training and treatment with *H. aphrodisiaca *for hydroxyproline levels in the tendon (p = 0.005).

### Zymography analysis

**Figure 3:**
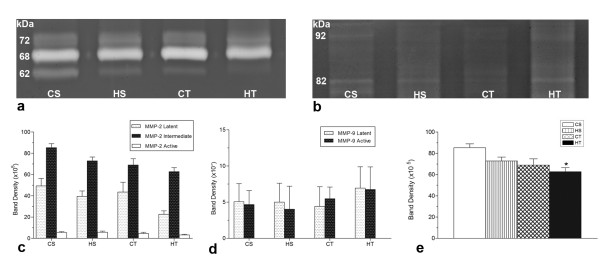
**Zymography of Achilles tendon extracts**. Each lane represents a sample of one animal. a, b- Gelatin zymography gel used to quantify activities of MMP-2 and MMP-9, respectively; c, d and e- Densitometry analysis of MMP-2, MMP-9 and total MMP-2, respectively. CS-control sedentary; HS- *H. aphrodisiaca *sedentary; CT- control trained; HT- *H. aphrodisiaca *trained. In the HT group the MMP-2 activity was reduced when compared with CS. MMPs activity was similar in both HS and CS groups, indicating the *H. aphrodisiaca *treatment did not inhibit MMP activity. MMP-9 latent (92 kDa), MMP-9 active (82 kDa), MMP-2 latent (72 kDa), MMP-2 intermediate (68 kDa), MMP-2 active (62 kDa). The columns are the mean ± SD. * Differences were significant for p < 0.05 (ANOVA) compared with CS group, by the Tukey test.

The zymography (Figure 3a and 3b) data demonstrated a tendency towards reduction of the activity of MMP-2 isoforms for the CT and HT groups (Figure 3c), but the difference between them was not statistically significant. The total MMP-2 activity was much reduced in the HT group (Figure 3e), when compared with other groups. MMP-9 activity was not statistically different for any of the experimental groups (Figure 3d).

### Morphology

**Figure 4:**
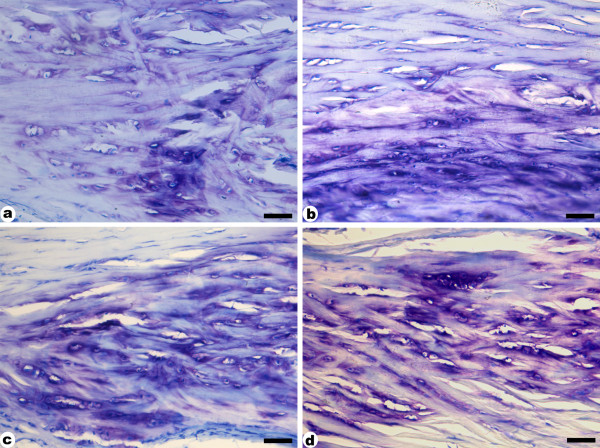
**Longitudinal sections of tendons stained with Toluidine Blue**. a- control sedentary; b- *H. aphrodisiaca *sedentary; c- control trained; d- *H. aphrodisiaca *trained. Observe intense metachromasy in trained groups (c, d). The treatment with *H. aphrodisiaca *accentuated metachromasy in sedentary (b) as well as trained (d) rats, compared with their respective controls. Scale bar = 25 μm.

In order to detect proteoglycans within the compression region of the tendon, sections of different experimental groups were stained with Toluidine Blue (Figure 4). Metachromasy was observed in the territorial matrix of the compression region of all groups as a violet coloring, which indicates higher concentrations of negative charges. The trained tendons (Figure 4c and 4d) had a larger and more intensely stained area compared to sedentary rats (CS and HS) (Figure 4a and 4b). This staining pattern suggests an increase in the proteoglycan concentration in this region.

**Figure 5:**
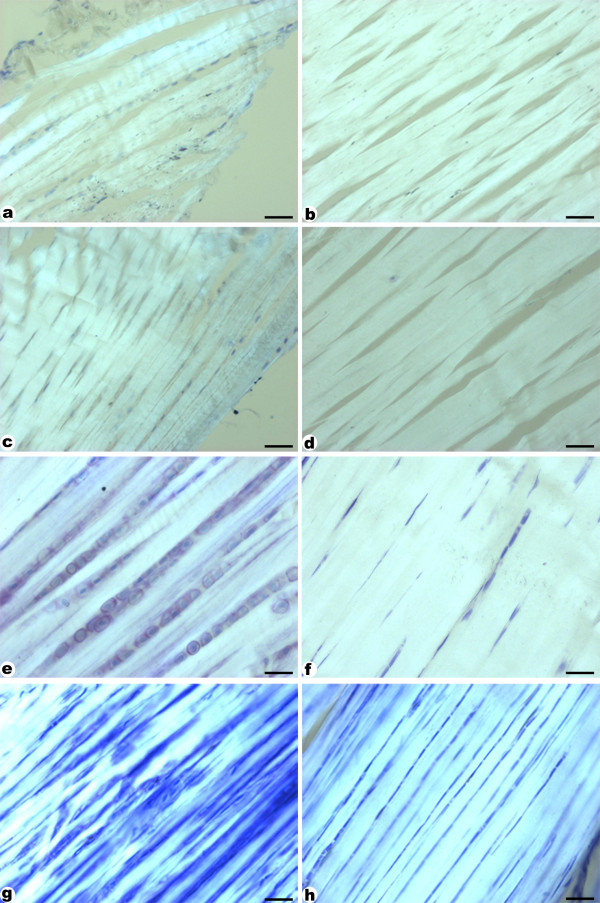
**Polarized light microscopy of Toluidine Blue-stained sections**. a, b- correspond to control sedentary; c, d- *H. aphrodisiaca *sedentary; e, f- control trained; and g, h- *H. aphrodisiaca *trained. The left column corresponds to the compression region and right column corresponds to the tension region. Observe a higher organization of the collagen bundles in tendons of trained (e-h) versus sedentary (a-d) animals. However, the most intense birefringence was found in tendons of trained rats and also those treated with the plant infusion (g, h). The stronger birefringence is due to the increased aggregation and longitudinal organization of the collagen bundles. Scale bar = 30 μm.

The analysis of trained tendons with polarized light microscopy showed intense birefringence in both compression (Figure 5e and 5g) and tension regions (Figure 5f and 5h), indicating highly organized collagen bundles. However, the HT group showed the most intense birefringence in both compression and tension regions of the tendon (Figure 5g and 5h). Both sedentary groups (Figure 5a, 5b, 5c and 5d) had the same morphological pattern and their collagen bundles were not as well aligned as observed in the trained ones.

### Biomechanical Parameters

**Figure 6:**
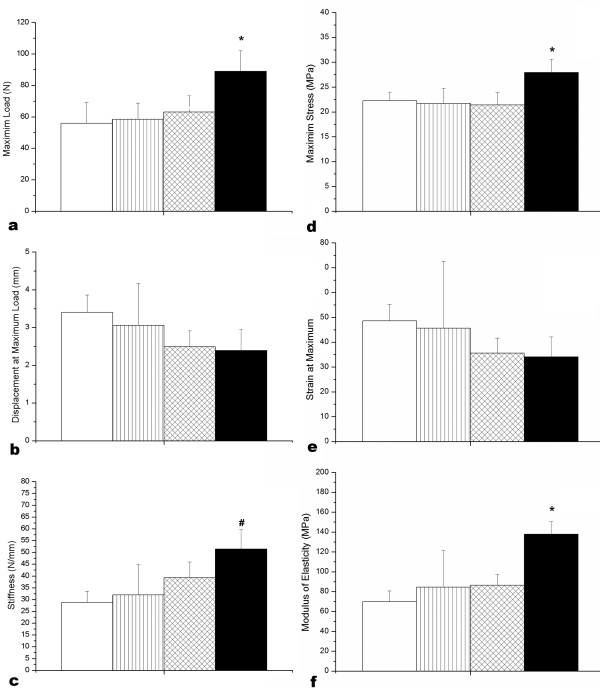
**Biomechanical properties of Achilles tendons from control and *H. aphrodisiaca *sedentary and trained rats**. The maximum load (a), stiffness (c), maximum stress (d) and modulus of elasticity (f), but not displacement (b) and strain (e), were higher in *H. aphrodisiaca *trained rats. CS- control sedentary; HS- *H. aphrodisiaca *sedentary; CT- control trained; HT- *H. aphrodisiaca *trained. The columns are the mean ± SD. * Differences were significant for p < 0.05 (ANOVA) compared with other groups, by the Tukey test. ^**# **^Differences were significant for p < 0.05 (ANOVA) compared with CS and HS, by the Tukey test.

The cross-sectional area increased 27% in HT tendons when compared with CS tendons (means: 3174.00 mm^2^±425.50 and 2493.2 mm^2^±373.14, respectively). The other groups did not show alterations in the tendon cross-sectional area. Biomechanical analysis showed significant increase in maximum load (Figure 6a), stiffness (Figure 6c), maximum stress (Figure 6d) and modulus of elasticity (Figure 6f) of the tendons of animals trained and treated with *H. aphrodisiaca *(HT). Two-way ANOVA analysis showed that there was interaction between training and treatment with plant infusion only for the maximum stress (p = 0.006). The displacement (Figure 6b) and strain (Figure 6e) were similar in all groups, with no significant difference among them.

## Discussion

The current study shows the biochemical, biomechanical and morphological alterations in Achilles tendon of rats concurrently submitted to endurance training and *H. aphrodisiaca *treatment.

The implications of endurance training [29-32] and AAS treatment [10,13,14] on tendon properties have been studied by several investigators. The use of AAS has resulted in a stiffer tendon that failed when submitted to less elongation than was achieved by the control Wistar rats. Exercise did not significantly alter tendon elongation in athlete animals. However, the combination of exercise and steroids significantly increased stiffness and decreased elongation, as well as the energy that the tendon could absorb at tendon failure [13]. In all cases the maximum load that the tendon could withstand did not seem to be affected. However, the use of steroids in the presence of exercise increased the cross-sectional area and reduced the flexibility of the tendon [13]. Wood et al. [10] in similar study, reported alterations in collagen content and the toe-limit strain in rats that had been treated with anabolic steroids and exercise.

In the present study, the maximum stress, modulus of elasticity and stiffness were higher in tendons of trained and treated rats, which also exhibited higher hydroxyproline content and increased cross-sectional area. These data showed that HT animals have more resistant tendons, differing from other studies in which the combination of AAS and exercise did not improve the tendon's biomechanical properties [10,13]. Moreover, the interaction between strenuous exercise and *H. aphrodisiaca *promoted significant increase in the material properties (maximum stress and modulus of elasticity) and of collagen content, resulting in stronger tendons able to support intense muscular contraction. Tendons may show a faster response to the number of loading cycles, rather than to the magnitude of the load [16]. Simonsen et al. [33] found that a strength-training regimen (high force with few loading cycles) did not stimulate increase in strength of the Achilles tendon of rats; however, low-force endurance training (e.g. swimming) resulted in stronger tendons. They suggested that the tendons may respond better to the number of muscle contractions that occur during training rather than the absolute tension exerted by the muscle. In this case, the increased tendon mechanical resistance observed during endurance training and *H. aphrodisiaca *treatment may represent a mechanism to prevent tendon damage due to mechanical fatigue. This biomechanical behavior could be due to the increase of the collagen content, to the fiber orientation and to the interaction between collagen and ground substance [34]. There is a relationship between mechanical properties and collagen content [16], since more resistant tissues have either more collagen per area or collagen fibers with larger diameters [34].

The biomechanical results corroborate the results obtained by polarized microscopy. The organizational aspect of the fibers is better understood when slides are analyzed under polarized microscopy, due to the birefringence properties of collagen bundles. This observation is important because it shows micro-morphological details hidden within these bundles. In the present study, this technique revealed high birefringent brightness due to the condensation and highly tidy fiber array in the trained group. Besides, in the trained group that also received the plant infusion (HT) the results were even more prominent, showing brighter collagen fibers, possibly indicating highly compacted bundles. The lower birefringence found in sedentary animals reflects less organized collagen bundles in these groups.

Some observations in the compression region showed that, in HT animals, there was an increase in the round cell population (stereological data not shown), as well as in the metachromasy intensity, which indicates a greater proteoglycan accumulation due to the increased compressive forces during endurance exercise. However, this result was not confirmed by the GAG dosage, which used the whole tendon. It is important to say that microscopical analysis of sections stained with toluidine blue specifically evaluate a region of the tendon where there is greater accumulation of proteoglycans due to the presence of localized compressive forces, as has been observed in tendons of rats [35] and pigs [36]. Nevertheless, proteoglycans, mainly the low weight ones, are distributed all over the tendon, associated to the collagen fibers [37], probably regulating collagen fibrillogenesis [38].

The degradation of collagen, as well as of a great number of other extracellular matrix compounds, is initiated by metalloproteinases (MMPs). An increase in net MMP activity is likely to indicate matrix degradation and accelerated remodeling [39,40]. Increased MMPs activity was verified in human tendons after acute running exercise [39] and in exercised rats (jumping in water) [14]. In the present study, zymography showed that MMP-2 activity in CT animals was similar in the sedentary groups, differing from other studies in which exercise increased the MMPs levels in tendons [14,39]. It is noteworthy that the MMP activity in the above studies was analyzed after only 3 days and 6 weeks of training, respectively.

In the present study, the animals were killed after 11 weeks of training, which means that the MMP activity was evaluated after a long training period. The biomechanical and morphological data demonstrated that the tendons of treated and trained animals had undergone adaptation to the increased demand. Thus, we suggest that there was a period of increased MMP activity to permit tissue remodeling, followed by a period of reduced activity when the tendons were already adapted to the load required. In this case, the mechanical load did not represent a stimulus for the synthesis of pro-MMPs and their eventual activation. Also, the MMP-9 activity did not alter in the exercised animals, which confirms the tendons' adaptation, since high activity of this protein is associated to the presence of immune cells during the initial inflammatory process [41], which occurs in response to tissue damage caused by exercise. In human tendons, protein synthesis and degradation were chronically elevated 4 weeks after the beginning of the training period, whereas protein synthesis remained high throughout a 12-week training cycle, while the degradation was slowly reduced. This suggests that there was probably an early period in the exercise program when collagen turnover in tendons was increased in order to restructure and readapt the tendon to the increased loading pattern [21].

Marqueti et al. [14] showed that the MMPs activity strongly decreased in AAS-treated animals. The inhibition could be due to a decrease in MMP synthesis or inhibition of activation of latent pro-MMPs. Also, that exercise by itself was not enough to compensate the inhibition of MMP activity induced by AAS treatment. In the present investigation the plant infusion without exercise did not inhibit MMP activity. The data obtained suggests that the infusion associated to exercise could have increased the MMPs' activity in the initial training period (1-3 weeks) when the highest mechanical loading occurred, considering that in the HT group the MMP-2 activity was reduced compared to all other groups, and that in this group the tendons were more resistant, according to the biomechanical results, in relation to the CT group. Therefore, we suggest that remodeling was more efficient in the HT group. However, further research is necessary to evaluate the effect of the plant infusion on MMP activity in exercised animals.

The present study faced some limitations regarding to the applied methodology, such as the lack of specific phytochemical data considering the active compounds present in *H. aphrodisiaca *infusion. Infusion preparation was based on previous studies that have been performed by our group using the same plant species [3,4,42], attempting to mimic the infusion traditionally used by folk medicine. Results of administering *H. aphrodisiaca *without exercise are not significantly different from the control, which would suggest a mandatory exercise program for efficacy of the plant extract altering rat tendons.

Common side effects due to long term endurance exercise together with synthetic AAS intake have been described as: collagen dysplasia, greater stiffness, reduction of strain, impaired tissue remodeling and others, none of which were noticed in this study after the training protocol and plant infusion administration. Scarce but important data lead us believe that there are no deleterious side effects related to *H. aphrodisiaca *intake, which was proved by evaluating blood biochemical parameters as well as kidney and liver morphology after long term administration of the plant infusion (data not published). Based on the promising data presented so far, future experiments crossing the variables of infusion dosages and time could be performed in order to potentiate *H. aphrodisiaca *effects along with endurance exercise. Additionally, phytochemical studies using the same infusion concentration are being carried out to reveal which active components could be related to the anabolic effects shown in this study.

## Conclusions

According to the results found in the present study, it can be concluded that endurance training associated with *H. aphrodisiaca *infusion increases the material properties of tendons. Rather, the association resulted in more resistant tendons, due to the increase in collagen molecules and the corresponding organizational increase. Despite the clearly anabolic effects of *H. aphrodisiaca *and associated endurance training, no side effects were observed, such as those found after synthetic AAS use. Therefore, *H. aphrodisiaca *associated with endurance training contributed to more efficient remodeling of the extracellular matrix, resulting in more resistant tendons to support high loads from intense muscle contraction. These findings suggest that *H. aphrodisiaca *infusion is a potential aid to optimize tendon remodeling in athletes, where the disparity of the faster physiological muscle adjustment in relation to the tendon often leads to lesions, because the tendons may not resist the increased tension produced by the stronger muscles.

## List of abbreviations

CS: control sedentary; HS: *H. aphrodisiaca *sedentary; CT: control trained; HT: *H. aphrodisiaca *trained; AAS: anabolic androgenic steroids; MMP: matrix metalloproteinase; GuHCl: guanidine chloride; PMSF: phenylmethylsulfonyl fluoride; EDTA: ethylenediamine tetraacetic acid; BSA: bovine serum albumin; GAG: glycosaminoglycans; DMMB: dimethylmethylene blue; CSA: cross-section area; NCP: noncollagenous proteins.

## Competing interests

The authors declare that they have no competing interests.

## Authors' contributions

JCM participated in all aspects of the work. The co-authors participated in the discussion of the results and revision of the manuscript, as well as in the following contributions: MLMG and MMS participated in the training program and the processing of the material; TCT participated in the morphological and biochemical analyses; WRN participated in the biomechanical analysis; DLF participated in the zymographical analysis; ERP and HD participated in supervision and as sponsors. All authors read and approved the final manuscript.

## Pre-publication history

The pre-publication history for this paper can be accessed here:

http://www.biomedcentral.com/1472-6882/11/51/prepub
